# Psychological Difficulties Mediate and Self-Efficacy Moderates the Relationship Between Family Cumulative Risk and Hope Among Chinese Children From Low-Income Families

**DOI:** 10.3389/fpsyg.2021.709320

**Published:** 2021-10-06

**Authors:** Xiayun Yin, Dongfang Wang, Zhihua Li, Yuesheng Huang

**Affiliations:** ^1^Department of Psychology, Hunan University of Science and Technology, Xiangtan, China; ^2^College of Psychology, South China Normal University, Guangzhou, China; ^3^Department of Psychology, Hunan First Normal University, Changsha, China

**Keywords:** family cumulative risk, psychological difficulties, self-efficacy, hope, children from low-income families

## Abstract

This longitudinal study investigated the role of psychological difficulties and self-efficacy in the relationship between family cumulative risk and hope among children from low-income families. The participants were 392 Chinese children from low-income families; the study extended for 2 years, and participants completed data that were collected with the following questionnaires: the Family Cumulative Risk Index, Children's Hope Scale, Strengths and Difficulties Questionnaire-Difficulties subscale, and General Self-efficacy Scale. The results demonstrated that psychological difficulties played a mediating role in the relationship between family cumulative risk and hope; specifically, family cumulative risk predicted hope of children via psychological difficulties. Self-efficacy moderated the relationship between psychological difficulties and hope. This moderation supported “a drop in the ocean effect”; the protective effect of high self-efficacy worked only when psychological difficulties were at low levels. When psychological difficulties were at high levels, the buffering effect of self-efficacy on family cumulative risk was gradually weakened and eventually lost.

## Introduction

In 1986, the Chinese government implemented a compulsory education policy—that it upholds to this day—providing education for grades 1 to 9 at no cost and requiring children to attend school through at least ninth grade. Nonetheless, many students drop out of junior high school each year. Economic factors have been regarded as a major cause for dropping out of school (Brown and Park, 2002). Indeed, poverty has been significantly correlated with dropout behavior (Mo et al., 2013; Wang et al., 2015), mainly owing to high tuition costs post-ninth grade in China. Among impoverished rural Chinese students, one study upheld that 14% of the students had left school in the first month of ninth grade (Yi et al., 2012). Moreover, low-income households have been associated with poor health and increased risk for mental health problems, and these can persist throughout childhood and adulthood of an individual (Hodgkinson et al., 2017). Children from low-income families generally experience higher rates of mental health problems and unmet mental health needs (Wadsworth and Achenbach, 2005). We, accordingly, felt this warranted an in-depth examination of the development and adaptation of Chinese children from low-income families; this knowledge may be useful for stakeholders to enhance support and intervention provisions, ensuring that these children have more opportunities to succeed in adulthood.

The concept of hope has been early theorized as a cognitive process concerning the desire of an individual to achieve clear and meaningful goals, generating motivation and strategies to ultimately achieve success (Snyder, 1995, 2002). This implies that systematic studies on hope among adolescents could yield important outcomes to the theoretic field and society. In adolescents, hope showed positive effects on one's academic and psychological aspects, academic achievement (Gallagher et al., 2017), and the buffering effects against negative psychological issues (Arnau et al., 2007). It also showed protective effects against school dropouts (Masten et al., 2008). Consequently, we rationalized that improving hope in children from low-income families could improve their academic development and protect their mental health.

The major influencing factor of hope in children is the family environment (Yin et al., 2019). In low-income scenarios, this factor may be further detrimental to children's hope development. Based on the family stress model, low-income families generally entail less-educated parents (Burlaka et al., 2015), parent–child communication, and parental participation (Asfour et al., 2017), and the adoption of negative parenting styles is not uncommon (Weitkamp and Seiffge-Krenke, 2019). Accordingly, children in low-income scenarios often face numerous adversities and multiple family risk factors simultaneously (Boe et al., 2018), and they commonly incur more adverse psychological outcomes (Burlaka et al., 2017).

Still, we thought that by focusing on low-income families, the multiple risk factors they may face, and their common effects, we could find ways to ensure the provision of greater consistency to children in these families. Specifically, a theory on the cumulative effect of risk factors on children suggests that a child's experience of a single risk does not necessarily lead to adverse developmental outcomes; however, upon experiencing multiple risk factors simultaneously, the probability of adverse outcomes greatly increases (Evans et al., 2013). Hence, we calculated the multiple family risks faced by children based on the cumulative risk model; this model proposes that, for a child's individual development, the total number of risk categories children face is more significant than the frequency and duration of a single risk factor (Evans et al., 2013). In this model, the calculation method requires each risk factor to be evaluated individually; if a specific risk is high, it is coded as 1, while if it is low or risk-free, it is coded as 0. Then, all risk factor scores are combined to determine the cumulative ecological risks next; the sum of all risk factors is totaled, facilitating the understanding and discussion of risk levels. The cumulative risk model showed a good prediction ability regarding the development outcomes of disadvantaged children (McLaughlin and Sheridan, 2016). Therefore, we deemed it of great value for our purpose to construct a multicomponent family cumulative risk (FCR) index and explore its impact on the hope of children.

The literature supports the idea that family risk can significantly predict adolescent psychological difficulties. In China, a cross-sectional study showed that FCR was associated with an increased emotion dysregulation and a decreased adaptive emotion regulation (Gao and Han, 2016). A cross-cultural study found a substantial connection between adolescent mental health and experiencing parental anxious rearing and psychological control (Weitkamp and Seiffge-Krenke, 2019). Previous studies have shown that emotional problems can significantly affect the level of hope of an individual for the future (Ouweneel et al., 2012). Hope is an important trait for children from low-income families to experience to face the future, and this feeling may be affected by their current level of psychological adaptation (Snyder, 2002). Hence, FCR seemingly influences the level of hope in children and the current and future mental health of a child.

Meanwhile, positive psychological resources, such as self-efficacy and self-esteem, could buffer the negative effects of ecological risk on the mental health of adolescents (Wright et al., 2013). Conceptually, self-efficacy refers to the overall confidence of an individual in the capacity to cope with various challenges/new situations (Schwarzer, 1994), being a major psychological influencing factor of hope levels (Snyder, 2002; Davidson et al., 2012). Corroborating, a study showed that high self-efficacy could promote confidence in the abilities of an individual, opportunity-seeking behavior for knowledge/skill improvement, and higher hope levels (Barrows et al., 2013). Moreover, self-efficacy, as a protective resource, is an important moderator in various settings (Heuven et al., 2006); exemplifying, individuals with high self-efficacy were more likely to adapt to/overcome risks or adversities, while those low in self-efficacy were more likely to incur adverse psychological outcomes (Bosmans et al., 2015). If an individual exhibits a high level of self-efficacy, he will have clearer goals and be able to achieve them more effectively. Resultantly, his level of hope would be higher. On the contrary, if the self-efficacy level of an individual is low, their goal will seem unclear. This may contribute to a lack of motivation in the pursuit of achieving his goal, resulting in a lower level of hope (Phan, 2013).

This study aimed to analyze the longitudinal relationship between FCR, psychological difficulties, self-efficacy, and hope in Chinese children from low-income families. We hypothesized that there would be a mediating effect of psychological difficulties on the relationship between FCR and hope and self-efficacy would play a moderating role in this model.

## Materials and Methods

### Participants

In November 2017, we conducted the baseline survey (Time 1, T1). Through convenience sampling, 504 seventh-grade children from low-income families were recruited; they came from 12 junior high schools in six cities in Hunan Province, China. In March 2018, participants from the T1 group were recruited for the follow-up survey (Time 2, T2). In November 2018, participants from the T2 group were recruited for the follow-up survey (Time 3, T3). In July 2019, participants from the T3 group were recruited for the final follow-up survey (Time 4, T4; losses to follow-up are reported in the Results section).

The inclusion criteria of the children were as follows: (1) being under 16 years of age at the baseline survey; (2) registered as a “Precision Student,” which is the local government's term for students coming from poor families; (3) have a family monthly income below 3,000 yuan; and (4) understand the questionnaire. The exclusion criteria were as follows: (1) students who had a major physical illness or (2) students who had a history of psychiatric conditions.

### Measures

#### Family Cumulative Risk

Concurring with previous research (Evans et al., 2013; Evans and Cassells, 2014), we selected six representative risk factors to evaluate FCR in our sample at T1, adopting classified variables (1 = risky, 0 = risk-free). Specifically, FCR covered family structure risk (1 factor), family resources risk (2 factors), and family atmosphere risk (3 factors); these factors were derived based on prior research (Buehler and Gerard, 2013).

Family structure risk was determined by family type. The situation was considered risky (i.e., coded as 1) when the child lived without their biological parents; otherwise, it was considered as risk-free (i.e., coded as 0; the coding for risk, 1, and risk-free, 0, was similar for all measures).

Family resource risk was determined based on parental education level and family economic stress. Parental education level was coded as 1 when parents had at or below a high school, technical secondary school, or vocational school education; parents with higher education levels were coded as 0. Family economic stress was assessed using the Economic Stress Questionnaire (Wadsworth and Compas, 2003), asking whether the family experienced economic stress in the past year. It was measured using four items (e.g., “My family doesn't have enough money to buy new clothes”), which were measured on a five-point Likert scale (1 = “*Never*,” 5 = “*Nearly always*”); higher scores reflect severe economic stress (Cronbach's α = 0.81). Upon calculating the total score of all items, we coded scores in the top quartile as 1; lower scores were coded as 0.

Family atmosphere risk was measured based on child–parent interaction, family function, and parental rearing style. Child–parent interaction was measured using one question: “How many hours per week do you spend interacting with your parents (e.g., playing and talking)?” It was measured on a four-point scale (1 = 0–3 *h*; 2 = 3–5 *h*; 3 = 5–7 h; and 4 = more than 7 h), and scores of 1 (i.e., 0–3 h per week) were coded as 1; otherwise, they were coded as 0.

Family function was measured using the Family APGAR Index (Smilkstein, 1984, 1993). It comprises five items addressing the following domains: adaptation, partnership, growth, affection, and resolve. This instrument uses a three-point Likert scale (0 = “*Rarely*” to 2 = “*Often*”), with lower total scores indicating lower family care levels (Cronbach's α = 0.80). Total scores of 7–10 indicated good family function, being coded as 0; total scores of 0–6 suggested moderate to severe family dysfunction, being coded as 1.

Parental rearing style was measured using the Parental Bonding Instrument (PBI; Mackenzie et al., 2011). It comprises two subscales: the mother rearing style (PBI-M, Cronbach's α = 0.78) and the father rearing style (PBI-F, Cronbach's α = 0.81). The 23 items in each subscale were measured on a scale ranging from 1 (“*Strongly disagree*”) to 4 (“*Strongly agree*”) regarding the described rearing style. Different total scores defined four types of parental rearing styles: authoritative, democratic, autocratic, and permissive (Parker et al., 1979). Adolescents experiencing either autocratic or permissive rearing styles were coded as 1; meanwhile, authoritative or permissive rearing styles were coded as 0.

#### Hope

Hope levels were measured at T1 and T4 using the Children's Hope Scale (CHS; Snyder et al., 1997). It comprises six items addressing two domains: agency and pathway thinking. The response scale ranged from 1 (“*None of the time*”) to 6 (“*All of the time*”), where higher scores indicated higher hope levels. The Chinese version of the CHS, upon being tested among Chinese children, showed adequate psychometric properties (Zhao and Sun, 2011). In our study, Cronbach's α was 0.79 at T1 and 0.84 at T4.

#### Psychological Difficulties

The Strengths and Difficulties Questionnaire-Difficulties subscale (SDQ-D) was used to assess the psychological difficulties of children for 6 months between T1 and T3. It addressed the following domains: hyperactivity-inattention (5 items), emotional symptoms (5 items), peer problems (5 items), and conduct problems (5 items) (Goodman et al., 1998). The response scale ranged from 0 (“*Not true*”) to 2 (“*Certainly true*”), and higher scores indicated a higher psychological difficulty. The Chinese version of the SDQ-D showed good applicability in the Chinese population (Yao et al., 2009). In our study, Cronbach's α was 0.86.

#### Self-Efficacy

Self-efficacy was measured using the General Self-Efficacy Scale (GSES), at T1, T2, and T3. The measure comprises 10 items, which are responded to on a four-point Likert scale; higher scores indicate higher self-efficacy (Cheung and Sun, 1999). In our study, Cronbach's α were 0.81, 0.78, and 0.83 at T1, T2, and T3, respectively.

### Procedure

At T1, all participants and their guardians received a standard letter of invitation. We obtained informed consent from all children and guardians before survey onset. Each participant and their guardians were also informed that they would receive a compensation of 50 RMB after survey completion. Students completed the questionnaire in a private meeting room, without any other students in it. One researcher remained in the meeting room with the student and answered the procedural questions. Participants were told that they could withdraw participation at any time, for any reason, and that they would be given contact information for relevant mental health services if they ever needed support. All children who partook in the survey at T1 were invited to participate in the follow-up surveys (i.e., T2–T4). According to the theoretical hypothesis of the introduction, we assessed cumulative ecological risk of children in T1, measured hope of children in T4, and measured the mediating variable (psychological difficulties), and moderator variable (self-efficacy) in T2 and T3. Because the surveys at T2 and T3 were conducted in spring and autumn, to avoid seasonal effects, we used the average values of mediating variables at T2 and T3.

### Statistical Analysis

The analyses were conducted using IBM SPSS Statistics version 23.0. We performed descriptive statistics to determine the sociodemographic characteristics and FCR. Then, we used Pearson's correlations to examine the associations between FCR and other variables of interest.

To examine common method variance before beginning regression analysis (Johnson et al., 2011), we conducted Harman's one-factor test. We tested the moderation and mediation hypotheses using PROCESS (Hayes, 2017). Bootstrap analysis was conducted with 5,000 iterations to estimate the size of the effects of each model, using 95% confidence intervals. First, we tested the mediation effect: we entered FCR at T1 (T1 FCR) as the predictor, the mean psychological difficulty scores at T2 and T3 (TM psychological difficulty) as the mediator, and hope at T4 as the outcome. We used the following control variables in the regression analysis: sex, psychological difficulties at T1 (T1 psychological difficulty), and hope at T1. Second, based on the mediation model results, we tested the moderated mediation hypotheses. We entered the mean self-efficacy scores at T2 and T3 as the moderator, while the self-efficacy score at T1 was used in the regression analysis as an additional covariate. Then, to explore the TM self-efficacy level at which the interaction between T1 FCR and TM psychological difficulties became significant, we calculated simple slopes for low TM self-efficacy (M-SD) and high TM self-efficacy (M+SD) separately. The statistical significance was set at *p* < 0.05.

## Results

### Sample Description

At T1, T2, T3, and T4, 504, 473, 419, and 392 children from low-income families completed the survey. The samples at each follow-up survey did not differ in sex, T1 FCR, T1 psychological difficulties, T1 self-efficacy, and T1 hope scores (*p* > 0.05 for all). At T4, the sample comprised 242 (61.7%) women and 150 (38.3%) men aged 11–15 years, with a mean age of 12.66 (SD = 1.23). Other sociodemographic characteristics are presented in Table 1.

**Table 1:** Descriptive sociodemographic variables.

**Sociodemographic variables**	**Note**	***n*(%)**
Age	Year[M(SD)]	12.66(1.23)
Gender	Female	242(61.7)
Place of residence	Rural	286(73.0)
Single-child family	Yes	66(16.8)
Ethnicity	Ethnic Han	319(81.4)
Family cumulative risk		
*Family structure risk*	(1) Living without the biological parents	88(22.4)
*Family resources risk*	(1) Low educational level of parents	259(66.1)
	(2) High family economic stress	116(29.6)
*Family atmosphere risk*	(1) Lack of interaction with parents	252(64.3)
	(2) Family with moderate or severe dysfunction	350(89.3)
	(3) Autocratic or permissive parental rearing styles.	130(33.2)

### Family Cumulative Risk Characteristics Among Chinese Children From Low-Income Families

Most children in our sample (89.3%) reported having a family with moderate or severe dysfunction, and more than 60% reported having parents with low education and lacking in parental interaction. The least prevalent family risk was living without biological parents (22.4%; see Table 1).

### Correlation Analysis

We found negatively significant correlations between hope and FCR scores; hope and psychological difficulties scores; self-efficacy, and FCR scores; and self-efficacy and psychological difficulty scores (Table 2). We also observed a significant positive correlation between hope and self-efficacy scores.

**Table 2:** Descriptive statistics and correlation analyses.

**Variables**	**M ± SD**	**1**	**2**	**3**	**4**	**5**	**6**	**7**	**8**
1.T1 Family cumulative risk	3.05 ± 1.18	-							
2,T1 Psychological-difficulties	31.66 ± 5.74	0.09	-						
3.T2 Psychological-difficulties	31.87 ± 5.68	0.14^**^	0.56^**^	-					
4.T3 Psychological-difficulties	32.30 ± 5.36	0.23^**^	0.43^**^	0.53^**^	-				
5.T1 Self-Efficacy	25.78 ± 5.02	−0.05	−0.32^**^	−0.24^**^	−0.19^**^	-			
6.T2 Self-Efficacy	26.24 ± 5.26	−0.11^*^	−0.29^**^	−0.30^**^	−0.22^**^	0.40^**^	-		
7.T3 Self-Efficacy	26.70 ± 4.65	−0.05	−0.23^**^	−0.26^**^	−0.37^**^	0.28^**^	0.36^**^	-	
8.T1Hope	21.86 ± 5.46	−0.02	−0.28^**^	−0.19^**^	−0.14^**^	0.64^**^	0.24^**^	0.02	-
9.T4 Hope	21.66 ± 5.56	−0.06	−0.19^**^	−0.26^**^	−0.27^**^	0.30^**^	0.36^**^	0.18^**^	0.29^**^

*T1: Time 1; T2: Time 2; T3: Time3; T4: Time4*;

* *p < 0.05*;

** *p < 0.001*.

### Mediation Analyses

After conducting an exploratory factor analysis (EFA), we found 14 factors with eigenvalues >1. Moreover, the first factor accounted for only 14.33% of the total variance. Hence, our study was not affected by the common method bias.

We conducted the mediation analysis using Model 4 in PROCESS. The T1 FCR scores did not directly predict T4 hope scores (*b* = 0.01, *p* = 0.17; Table 3). However, the main effect of T1 FCR and TM psychological difficulties scores was significant and was positively associated (*b* = 0.17, *p* < 0.01). There was also a significantly negative effect of TM psychological difficulties on T4 hope (*b* = −0.27, *p* < 0.01). Additionally, when controlling for sex, T1 hope, and T1 psychological difficulties, the indirect effect of TM psychological difficulties on the relationship between T1 FCR and T4 hope scores was significant (*b* = −0.05, 95% CI = −0.08 ~ −0.02). Therefore, psychological difficulties fully mediated the effects of FCR on hope among children from low-income families in our sample.

**Table 3:** Regression results of testing the mediating model.

**Variables**		**Step 1**				**Step 2**		
	***b***	**S.E**.	***t***	**95%CI**	***b***	**S.E**.	***t***	**95%CI**
Gender	0.13	0.09	1.44	−0.05,0.31	−0.26	0.10	−2.56^**^	−0.47, −0.06
T1 Hope	0.01	0.05	0.12	−0.08,0.10	0.24	0.05	4.72^**^	0.14,0.35
T1 Psychological difficulties	0.53	0.05	11.32^**^	0.44,0.63	0.01	0.06	0.17	−0.11,0.13
T1 Family cumulative risk	0.17	0.05	3.84^**^	0.08,0.26	0.01	0.05	0.16	−0.09,0.11
TM Psychological difficulties					−0.27	0.06	−4.57^**^	−0.41, −0.16
*F*		42.34^**^				14.82^**^		
*R^2^*		0.34				0.19		

*T1: Time 1; T2: Time 2; T3: Time3; T4: Time4; TM: mean value at T2 and T3; Step 1: the predicted variable is TM psychological difficulties; Step 2: the predicted variable is T4 Hope*;

* *p < 0.05*;

** *p < 0.001*.

### Moderation Analyses

To further reveal the moderating effect of self-efficacy between psychological difficulty and hope, the positive and negative standard deviations of the mean value of self-efficacy were taken as the dividing point. The participants with scores higher than one standard deviation were classified as high self-efficacy group, while those with scores lower than one standard deviation were classified as low self-efficacy group. The specific moderating effect is shown in **Figure 2**.

**Figure 1:**
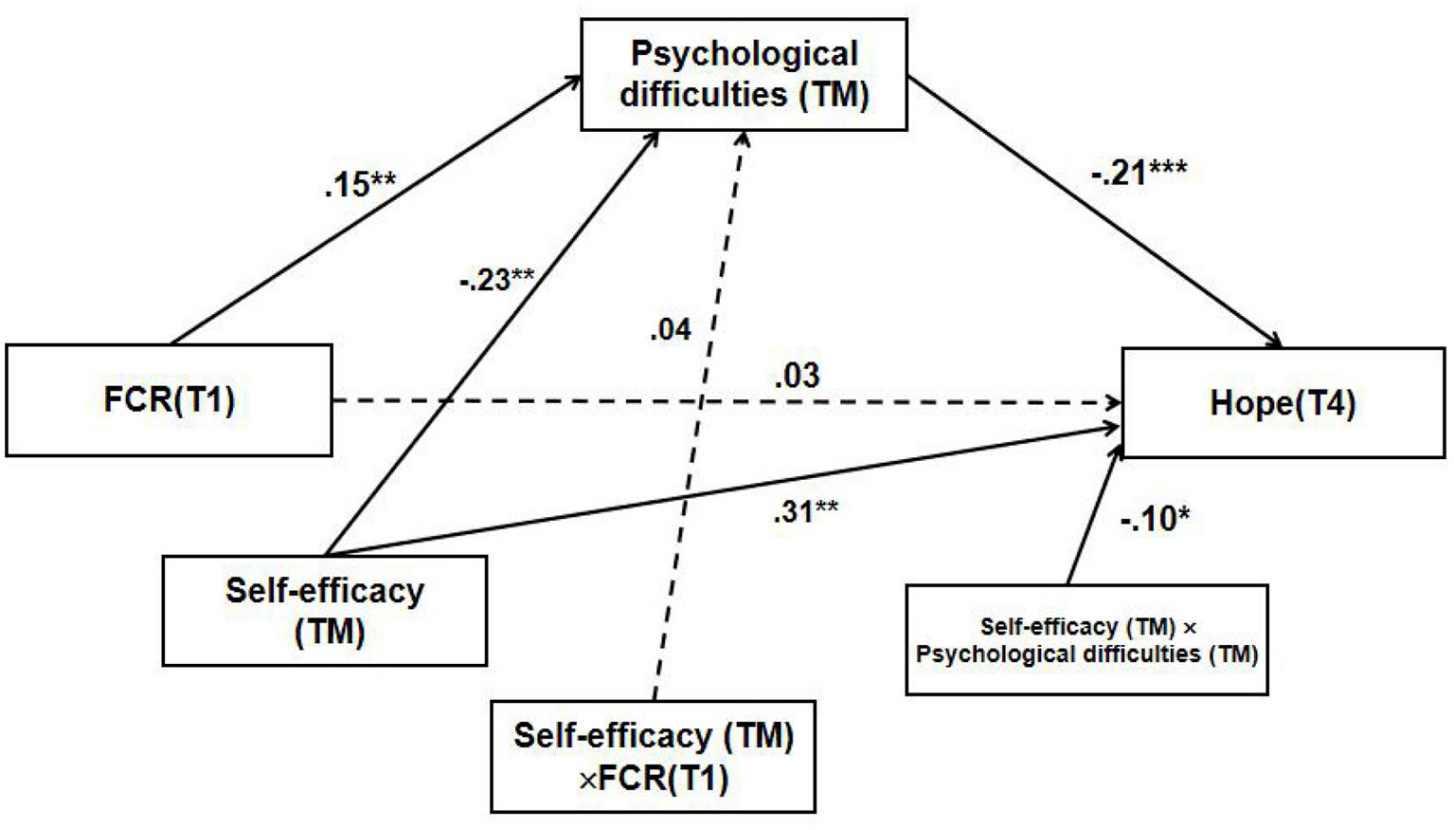
Results of the moderated mediation analysis. FCR, Family cumulative risk, *p > 0.05, **p < 0.01, ***p < 0.001.

**Figure 2:**
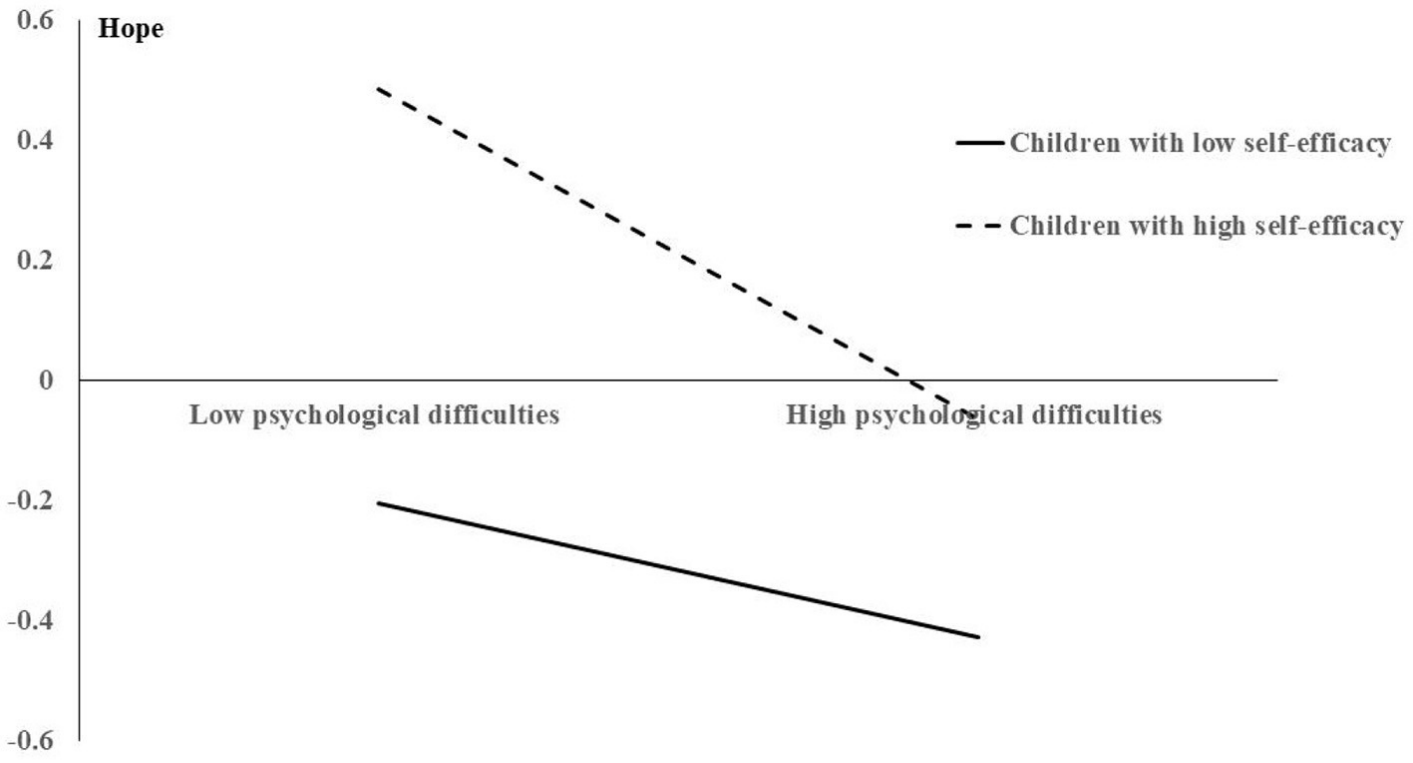
The interaction between self-efficacy and psychological difficulties in predicting hope.

The moderation model was significant, with self-efficacy as the outcome (Figure 1). Only the interaction between self-efficacy and psychological difficulties predicted T4 hope in the moderated mediation models. Hence, self-efficacy moderated the relationship between T1 FCR and T4 hope. This implies that the indirect effect of T1 FCR on T4 hope, mediated by TM psychological difficulties, significantly differed by self-efficacy. We observed, through simple slope analyses, that there was a weak negative relationship between TM psychological difficulties and T4 hope at a low level of self-efficacy (*b* = −0.12, SE = 0.07, *p* = 0.09, 95% CI = −0.26, 0.02). Meanwhile, we found a strong significant negative relationship at high levels of self-efficacy (*b* = −0.29, SE = 0.08, *p* < 0.001, 95% CI = −0.46, −0.13; Figure 2).

## Discussion

### Family Cumulative Risk Status Among Chinese Children From Low-Income Families

Previous studies have found that children from low-income families face twice as many risks as the general population (Anda et al., 2010), raising their risk index for emotional and behavioral problems two to five times more than that of other children (Freeman and Pamela, 2014). Therefore, when examining the impact of poverty on the mental health of children, we must also consider the cumulative effects of other risk factors. Our results showed that most Chinese children from low-income families experienced family risk; this concurs with existing research (Burlaka et al., 2015; Asfour et al., 2017; Weitkamp and Seiffge-Krenke, 2019). Moreover, our findings suggested that our sample often faced multiple and cumulative family risk factors simultaneously. Compared with the general population, the risk of family resources related to the economy is particularly serious for children in low-income families. Due to low education levels, it is difficult for parents to obtain high-income or stable jobs, resulting in less investment in the upbringing of children. Additionally, the living environment needs improvement (Gao and Han, 2016; Boe et al., 2018). We hypothesized that the impact of family poverty on parents migrated into the parent–child interactions. Indeed, a study showed that the risk of economic stress is likely to affect the psychological adaptation of parents, resulting in various increased risk factors in the familial experiences of the child, such as family care, parental marital quality, and parenting style (Masarik and Conger, 2017). Therefore, to understand the situations of children in low-income families, stakeholders should observe FCR of children and the possible effects on the child.

### The Relationship Between FCR, Psychological Difficulties, and Child Hope

The most striking finding of this study was that psychological difficulties fully mediated the relationship between FCR and hope during the entire 2-year study period. Additionally, FCR had a positive effect on psychological difficulties. Previous studies have suggested that in poor families, parents must cope with continuous survival and economic pressures, diminishing their energy and patience, thereafter, hindering their ability to pay attention to and meet the emotional needs of their children. Then, when children do not have their normal material and emotional needs relevantly met, they often become stressed and insecure, leading to psychological difficulties (e.g., emotional problems) (Evans and Kim, 2013). Meanwhile, regarding hope, early theories have suggested that hope is not innate; it is acquired and altered through learned experiences (Snyder et al., 1991). Hence, we speculated that psychological difficulties in children could cause them to experience stress; thereafter, stress reduces positive psychological resources of children, finally inhibiting the development of hope. Our results supported the past research (Fredrickson, 2001), which has demonstrated that when children experience more positive emotions, they become more focused and open; the study describes that, in this state, children feel an urge to try new approaches, develop new problem-solving strategies, and make creative efforts. Another study found that the pathways between depression and hope are significant (Li et al., 2018). To summarize, by encouraging individuals to think positively about the possible outcomes of various actions, positive emotions can expand the scope of attention, cognition, and action of an individual; this may allow individuals to achieve a higher level of pathway thinking. Positive emotions can also strengthen the lasting physical and psychological resources of an individual, giving them stronger motivation to pursue goals and achieve a higher level of agency thinking.

Moreover, positive family environments showed a significant impact on an orientation of a child toward and planning of future goals; specifically, children from families with low family risk tended to have more positive and clear future goals, whereas their counterparts from families with high family risk tended to have negative and vague future goals (Griskevicius et al., 2011; Schroder et al., 2011). From this, we can infer that FCR has a direct impact on children's development of hope. Contrarily, our findings showed that FCR did not significantly predict the long-term hope. This could be because most children in our sample spent most of their time at school. When constantly separated from the family environment, over time, its impact may gradually weaken. Hence, the school environment, where many Chinese children spend most of their time, may have a greater influence on the children's development of hope. Concurrently, poverty may affect children's levels of satisfaction and connection with their school, affecting the quality of their interpersonal communication and influencing their mental health (Clements-Nolle and Waddington, 2019; Saunders and Brown, 2020).

### A Drop in the Ocean Effect of Self-Efficacy

Our evidence showed that high self-efficacy was associated with decreased psychological difficulty levels and elevated hope levels. This concurs with the literature, which indicated that self-efficacy significantly predicts mental health; specifically, individuals with high self-efficacy tended to have higher levels of positive mental health factors (e.g., life satisfaction, self-esteem, and positive emotions) and lower levels of negative factors (e.g., loneliness, depression, anxiety, and negative emotions) (Jiang et al., 2015; Gao et al., 2017; Wang and Zhang, 2017). Self-efficacy was also shown to have a significant positive effect on hope, and individuals with high self-efficacy had clearer goals and achieved them more effectively (Snyder, 2002; Phan, 2013).

Our findings showed that self-efficacy moderated the association between psychological difficulties and hope; specifically, this moderation supported a prior notion: the “drop in the ocean effect” (Li et al., 2012). Namely, the protective effect of high self-efficacy worked only when psychological difficulties were at low levels. When psychological difficulties were at high levels, the buffering effect of self-efficacy on FCR was gradually weakened and eventually lost. Based on prior research (Anderson et al., 2006; Wiederkehr et al., 2015), we believe that, in children from low-income families, a higher level of FCR may lead to more psychological difficulties and also hinder self-efficacy development.

This understanding allows us to speculate that most Chinese children in low-income families are low in self-efficacy; if accurate, this supposition entails that in such poverty settings, only children with low individual psychological risk levels may gain any protection through self-efficacy. Therefore, when external or internal psychological risks are too high in children from low-income families, interventions focused on increasing positive psychological aspects may not provide a sustainable impact on their psychological state.

### Clinical Consideration

According to the results of this study, the following considerations can be made when designing positive interventions to promote the level of hope in children from low-income families:

It has been demonstrated that the cumulative family risk affects the level of hope in children from low-income families. Therefore, we can consider reducing family risk factors to increase levels of hope in children. For example, parents may be advised to devote more time to parent–child communication. Second, we may also begin with an intervention for the psychological difficulties that children in low-income families face. We can mitigate the negative impact of family risk on children's future levels of hope by improving their mental health levels. Finally, the “a drop in the ocean” effect of self-efficacy reminds us that while we strive to improve children's self-efficacy, we must also recognize the limitations of the protective role of self-efficacy and pay attention to implementing interventions that help reduce family risk.

### Limitations

First, the sample shedding rate in our study was relatively high, which may have biased the results. One primary reason for the sample attrition was that the parents of many Chinese children from low-income families are often migrant workers; hence, their children often have high mobility and school transfer rates. These characteristics hindered our ability to retain the sample for the follow-up surveys. Second, the reported psychological difficulties were measured through self-reported questionnaires; these are subject to the influence of social desirability. Third, we believe that important co-occurring risk factors (e.g., peer relations or social support networks), which have effects that cannot be analyzed via statistical analyses, may have influenced our findings. Finally, there was no longitudinal survey of children from middle-income families for comparative analysis in our study.

## Conclusions

The present longitudinal study was the first to support the potential moderating (self-efficacy) and mediating (psychological difficulties) factors of the relationship between FCR and hope among children from low-income families in China. Our findings suggested that psychological difficulties mediate the aforementioned relationship; namely, FCR seemingly predicted hope levels via psychological difficulties in our sample. Self-efficacy, meanwhile, served as a moderator between psychological difficulties and hope. Specifically, self-efficacy buffered the impact of psychological difficulties on hope, albeit the buffering effect was smaller if psychological difficulties were higher. Overall, family risk factors and psychological difficulties were shown to be key predictors of hope among Chinese children from low-income families. Hence, it is important not to be overly optimistic about the protective role of self-efficacy for children in such settings.

## Data Availability Statement

The raw data supporting the conclusions of this article will be made available by the authors, without undue reservation.

## Ethics Statement

The studies involving human participants were reviewed and approved by Ethics Committees of Hunan Key Laboratory of Children's Psychological Development and Brain Cognitive Science, Hunan First Normal University. Written informed consent to participate in this study was provided by the participants' legal guardian/next of kin.

## Author Contributions

XY and YH was mainly responsible for the overall conception and design of this study. DW wrote the manuscript and carried out the statistical analysis. ZL carried out the investigation and data collation work. YH carried out the language polishing for the manuscript. All authors contributed to the article and approved the submitted version.

## Funding

This work was supported by Scientific research project of Hunan Education Department (17K049). A key funded project of Hunan Educational Science Planning in 2019 (XJK19AXL003).

## Conflict of Interest

The authors declare that the research was conducted in the absence of any commercial or financial relationships that could be construed as a potential conflict of interest.

## Publisher's Note

All claims expressed in this article are solely those of the authors and do not necessarily represent those of their affiliated organizations, or those of the publisher, the editors and the reviewers. Any product that may be evaluated in this article, or claim that may be made by its manufacturer, is not guaranteed or endorsed by the publisher.
